# Clinical Applications of TSPO PET for Glioma Imaging: Current Evidence and Future Perspective—A Systematic Review

**DOI:** 10.3390/diagnostics13101813

**Published:** 2023-05-21

**Authors:** Luca Filippi, Viviana Frantellizzi, Giuseppe De Vincentis, Orazio Schillaci, Laura Evangelista

**Affiliations:** 1 Nuclear Medicine Unit, “Santa Maria Goretti” Hospital, Via Antonio Canova, 04100 Latina, Italy; 2 Department of Radiological Sciences, Oncology and Anatomo-Pathology, Sapienza, University of Rome, 00185 Rome, Italy; 3 Department of Biomedicine and Prevention, University Tor Vergata, Viale Oxford 81, 00133 Rome, Italy; 4 Nuclear Medicine Unit, Department of Medicine (DIMED), University of Padua, Via Giustiniani, 35128 Padua, Italy

**Keywords:** gliomas, translocator protein, TSPO, molecular imaging, PET/CT, theranostics

## Abstract

Our aim was to provide a comprehensive overview of the existing literature concerning the clinical applications of positron emission computed tomography (PET) with radiopharmaceuticals targeting the translocator protein (TSPO) in gliomas. A literature search for studies about TSPO PET in the last 10 years (from 2013 to February 2023) was carried out on PubMed, Scopus, and Web of Science using the following keywords: “PET” AND “Gliomas” AND “TSPO”. The Critical Appraisal Skills Program checklist for diagnostic test studies was used for testing the quality of selected papers. Ten articles were selected, encompassing 314 glioma patients submitted to PET/CT (9/10) or PET/MRI (1/10) with TSPO ligands. Among the various available TSPO tracers, the most frequently used was the third-generation ligand, [^18^F]-GE-180. TSPO PET results were useful to identify anaplastic transformation in gliomas and for the prognostic stratification of patients bearing homogeneous genetic alterations. When compared to amino-acid PET, TSPO PET with [^18^F]-GE-180 presented superior image quality and provided larger and only partially overlapping PET-based volumes. Although biased by some issues (i.e., small sample size, most of the studies coming from the same country), preliminary applications of TSPO PET were encouraging. Further studies are needed to define implications in clinical practice and shape the role of TSPO PET for patients’ selection for potential TSPO-targeted molecular therapies.

## 1. Introduction

Gliomas are the most frequent primary malignant tumors: in spite of the many advances in diagnosis, their mortality and recurrence rate still remain high and the prognosis is dismal, which is also due to the limited therapeutic options [1].

Conventional magnetic resonance imaging (MRI) is the gold standard for diagnosis and follow-up, although it presents some limitations for the pre-operative characterization of tumor grade (high- vs. low-grade gliomas) and for the differentiation of treatment-related changes from tumor recurrences (i.e., radionecrosis and pseudoprogression) [2]. Although the aforementioned limitations have been partially overcome by the implementation of magnetic resonance spectroscopy (MRS), a valuable tool to quantify tumor-associated metabolites, there is still an unmet need for imaging biomarkers suitable for improving glioma in vivo pre-operative characterization and for monitoring the response to therapy [3].

Positron emission tomography (PET/CT) with radiolabeled amino acids, such as ^11^C-methyl-l-methionine ([^11^C]MET), O-(2-[^18^F]fluoroethyl)-l-tyrosine ([^18^F]FET), and 3,4-dihydroxy-6-[^18^F]-fluoro-l-phenylalanine ([^18^F]-FDOPA), has been successfully implemented in neuro-oncology, particularly to discriminate radionecrosis/pseudoprogression and tumor recurrence after surgery and radiochemotherapy [4,5,6,7,8,9]. Notably, the World Health Organization (WHO) Classification of Tumors of the Central Nervous System has further highlighted the relevance of molecular profiling, especially methylation of O⁶-methylguanine-DNA methyltransferase promoter (MGMTp) and isocitrate dehydrogenase 1 (IDH 1) mutations, for categorization and prognostication of brain tumors [10].

In recent years, the translocator protein (TSPO), an 18 kDa protein mainly expressed on the outer mitochondrial membranes of microglia, astrocytes, and endothelial cells, has been catalyzing attention as a relevant biomarker of neuroinflammation. Although its role is still not completely understood, TSPO seems to be involved in transporting cholesterol into mitochondria, in steroid synthesis, and in other bioenergetics reactions [11]. Notably, TSPO has been found strongly up-regulated when microglia are activated as a response to injury, therefore, it has been emerging as an interesting biomarker for several neuroinflammatory and neurodegenerative conditions (e.g., multiple sclerosis, Alzheimer’s disease, etc.) [12,13,14]. On the other hand, aside from being overexpressed on activated microglia, an increased TSPO expression has been reported on glioma tumor cells, with a positive correlation with tumor grade and biological aggressiveness [15,16]. In this perspective, TSPO might represent a valuable biomarker for simultaneously assessing glioma biological characteristics as well as having insight into the glioma-associated tumor microenvironment (TME).

Several efforts have been made to synthesize TSPO ligands suitable for PET imaging. [^11^C]-PK11195, a selective antagonist for TSPO, has been first investigated as an imaging agent with encouraging results, although its strong binding to plasma protein and relatively low brain permeability led to a sub-optimal signal-to-background ratio. In addition, the ^11^C short half-life restricted its employment in clinical centers equipped with an in situ cyclotron. Subsequently, second-generation TSPO ligands, such as [^11^C]-DPA-713 and the ^18^F-derivative [^18^F]-DPA-714, have been introduced with meaningful improvement of image quality, and, even more recently, the third-generation TSPO ligand, [^18^F]-GE-180 was developed and exhibited superior imaging properties [17]. Nevertheless, the affinity of TSPO ligands to the target is deeply influenced by a single polymorphism (rs6971) in exon 4 of the TSPO gene, according to which patients can be stratified into high-affinity, medium-affinity, and low-affinity binders [13].

The aim of this present systematic review is to provide a comprehensive overview of the existing literature concerning the clinical applications of PET with TSPO ligands (i.e., TSPO PET) for glioma imaging, also trying to delineate the next steps for its implementation in clinical practice.

## 2. Materials and Methods

### 2.1. Search Strategy

A literature search until February 2023 was performed in PubMed, Web of Science, and Scopus databases in order to retrieve papers related to the topic, according to the Preferred Reporting Items for Systematic reviews and Meta-analyses (PRISMA) guidelines [18]. The terms used were: “PET” AND “Gliomas” AND “TSPO”. The following types of studies were considered: head-to-head comparative series, matched-pair studies, clinical trials, case series, prospective studies, and retrospective cohorts. Case reports, review papers, conference proceedings, editorial commentaries, interesting images, and letters to the editor were excluded. Only studies published from 2013 up to February 2023, limited to humans, and in the English language were selected.

Two reviewers (L.F., L.E.) conducted the literature search and independently appraised each article using a standard protocol and data extraction. The reference lists of the selected studies were carefully checked to identify any additional relevant literature.

From each study extracted data were, respectively: type of the study (prospective, retrospective, etc.); year and location of the study; sample size; clinical setting; utilized tracer and administered activity; utilized device (PET/CT or PET/MRI); standard of truth (i.e., histopathology, clinical follow-up, or other). Studies with incomplete technical or clinical data were considered ineligible.

### 2.2. Quality of the Selected Studies

Selected imaging studies were analyzed using a modified version of the Critical Appraisal Skills Programme (CASP) (https://casp-uk.net/aboutus, accessed on 1 January 2023) checklist for diagnostic test studies. Critical appraisal was performed by 2 reviewers (L.F. and L.E.), and discrepancies, if any, were solved by discussion with the other authors (G.D.V., O.S., and V.F.).

## 3. Results

### 3.1. Analysis of the Evidence

**Figure 1:**
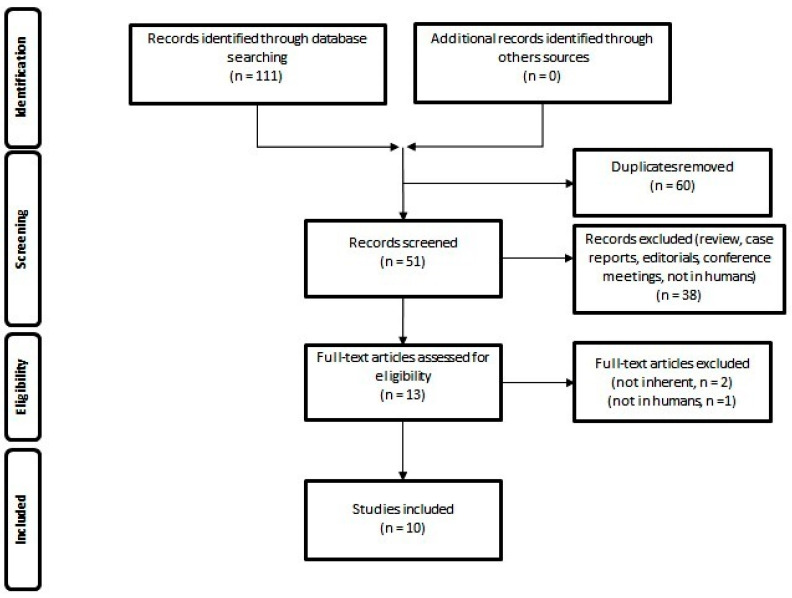
Schematic representation of PRISMA workflow for manuscripts’ selection.

The resulting PRISMA search strategy is shown in Figure 1. From the systematic literature search, 10 papers were selected, for an overall number of 314 patients affected by gliomas and submitted to PET with various TSPO ligands. Table 1 summarizes the main findings of the selected manuscripts.

From the analysis of the selected papers, we identified three main thematic areas: (1) correlative studies between TSPO PET findings and glioma tumor grading, molecular profiling, or post-recurrence survival; (2) comparative studies between TSPO PET and amino-acid PET and/or MRI; (3) kinetics, methodological, or feasibility studies.

In the majority of the selected papers, [^18^F]-GE-180 was employed, [^11^C]-(R)PK11195 was utilized in two studies and [^18^F]-DPA-714 was reported in one manuscript. As far as it concerns the various employed tomographs, in seven studies PET/CT was utilized, in two papers an emissive research-dedicated PET was employed, and a ^123^Cs-source was used for scatter and attenuation correction, only one paper utilized a PET/MRI tomograph. PET was carried out both through dynamic and static acquisition modalities; for [^18^F]-GE-180 tracer a time window of 60–90 min post-injection (p.i.) was the optimal timing for obtaining high-quality images.

**Figure 2:**
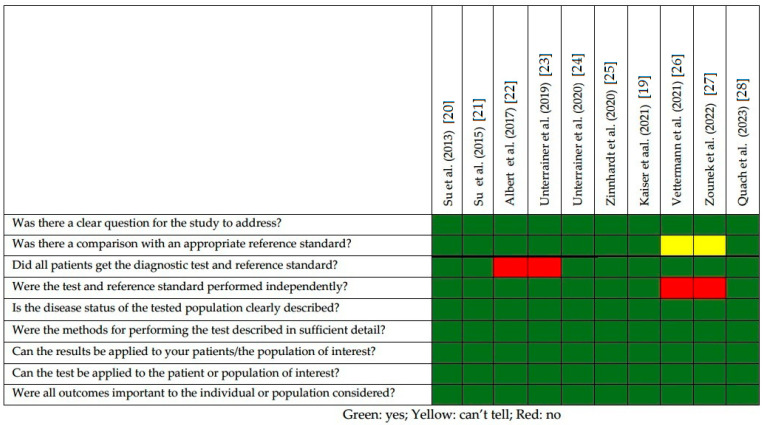
Quality appraisal of selected articles using CASP checklist for diagnostic studies [20,21,22,23,24,25,26,27,28].

The quality appraisal of the selected studies is represented in Figure 2. The majority of studies had neuropathology as a reference, also encompassing histochemical analysis for determining TSPO expression on glioma and glioma-infiltrating cells. The most relevant limitations in the selected studies were the following: (1) small sample size, exceeding the threshold of 50 patients only in two studies (20%); (2) the majority (80%) of the selected studies were carried out in Germany and 70% of the papers were performed by the same group of researchers. In this regard, in one manuscript, the authors specified that they included in their cohort patients collected from previously published reports (5 out of 34 enrolled subjects, 14.7%) [19].

The findings of the selected papers for each thematic area are described in the following paragraphs.

**Table 1:** Main findings of the selected papers on TSPO PET applications in gliomas.

References	Year/Location	Study	Setting	Sample Size	Tracer	Methodology	SoT
Su et al. [20]	2013/UK	Feasibility	Therapy “naïve”	23	[^11^C]-(R)PK11195	Activity: 517 ± 127 Device: PET Scan modality: dynamic (60 min in list mode as 18 time-frames)	NPath
Su et al. [21]	2015/UK	Prospective	Prognostic stratification	22	[^11^C]-(R)PK11195	Activity: 509.6 ± 123 MBq Device: PET Scan modality: dynamic (60 min in list mode as 18 time-frames)	NPath
Albert et al. [22]	2017/Germany	Pilot Study	Pre-operative characterization of recurrence	11	[^18^F]-GE-180	Activity: 187 ± 13 MBq Device: PET/CT Scan modality: dynamic in 1 case, static with a 20-minute duration at 60 min p.i. in 10 pts	NPath or follow-up
Unterrainer et al. [23]	2019/Germany	Pilot study	Pre-operative or recurrence	20	[^18^F]-GE-180	Activity: 178 ± 12 MBq Device: PET/CT Scan modality: data acquired at 60–80 min p.i.	NPath or follow-up
Unterrainer et al. [24]	2020/Germany	Pilot study	Pre-operative or recurrence	58	[^18^F]-GE-180	Activity: 185 ± 14 MBq Device: PET/CT Scan modality: data acquired at 60–80 min p.i.	
Zinnhardt et al. [25]	2020/Germany	Prospective	Pre-operative or recurrence	9	[^18^F]-DPA-714	Activity: 247.3 ± 27.3 MBq Device: PET/MRI Scan modality: dynamic acquisition in 60 min	Npath and immunophenotyping
Kaiser et al. [19]	2021/Germany	Prospective	Pre-operative (newly diagnosed)	34	[^18^F]-GE-180	Activity: 185 ± 14 MBq Device: PET/CT Scan modality: data acquired at 60–80 min p.i.	NPath
Vettermann et al. [26]	2021/Germany	Feasibility	Therapy “naïve”	30	[^18^F]-GE-180	Activity: 189 ± 12 MBq Device: PET/CT Scan modality: data acquired at 60–80 min p.i.	N.A.
Zounek et al. [27]	2022/Germany	Feasibility	Pre-operative or recurrence	19	[^18^F]-GE-180	Activity: 172 ± 11 MBq Device: PET/CT Scan modality: dynamic acquisition in 90 min, the 60–90 min summed image used for analysis	N.A.
Quach et al. [28]	2023/Germany	Retrospective, observational	Recurrence	88	[^18^F]-GE-180	Activity: 180 MBq Device: PET/CT Scan modality: data acquired at 60–80 min p.i.	Follow-up (post-recurrence survival and time to treatment failure).

SoT: standard of truth; NPath: neuropathology; N.A. = not available.

### 3.2. Correlation with Tumor Grading, Molecular Profiling, and Post-Recurrence Survival

Su et al. investigated the potential usefulness of TSPO PET with [^11^C]-(R)PK11195 to differentiate low-grade (LGGs) from high-grade gliomas (HGGs), also in the perspective of eventual TSPO-targeted therapies [21]. The authors enrolled patients affected by brain lesions with MRI features typical for grade II or III gliomas according to the WHO classification. In addition, two subjects with MRI features typical for GBM were also included in the study, for comparison. In all cases, both MRI and PET scans were acquired. In particular, PET was carried out through dynamic acquisition, each summed PET image was co-registered with the corresponding MRI image. Parametric maps representing the tracer binding potential (BP_ND_ = ratio of specifically bound radioligand over the non-displaceable one, using cerebellum as a reference region) were generated. Image analysis was correlated with post-surgical neuropathological findings: 13 astrocytomas (6 grade II, 4 grade III, 3 grade IV) and 9 oligodendrogliomas (7 grade II, 2 grade III) were identified. At MRI, contrast-enhancing was observed in the two GBM, while it was almost absent in other tumors, while perfusion analysis (rCBV) showed substantial heterogeneity among the various lesions. On PET imaging, LGGs were characterized by significantly lower tracer BP_ND_ with respect to HGGs. Notably, histochemical examination demonstrated that TSPO was expressed predominantly on the tumor cell surface and only in a lesser extension on the glioma-associated microglia/macrophages (GAMs), with a positive correlation between BP_ND_ gauged on PET images and TSPO density on histological specimens. The aforementioned results supported the potential employment of TSPO PET for the pre-operative detection of anaplastic transformation in gliomas. In addition, since initially performed studies seemed to suggest that the uptake of TSPO ligands might depend more on the blood–brain barrier (BBB) disruption than on the specific tracer binding to the target, the authors underlined how, in their cohort, [^11^C]-(R)PK11195 was detected also in HGGs characterized by no or minimal BBB damage.

Unterrainer and collaborators investigated the correlation between TSPO PET with [^18^F]-GE-180 PET and glioma biological characteristics in 58 patients affected by newly diagnosed (*n* = 33) or recurrent (*n* = 25) brain tumors [24]. After injection of 180 MBq of tracer, PET/CT acquisition was obtained at 60–80 min p.i. The following PET-derived parameters were calculated: standardized uptake value (SUV) of background activity (SUV_BG_); maximum and mean tumor-to-background ratio (TBR_max_ and TBR_mean_); and PET-volumes semi-automatically delineated using a threshold of SUV_BG_ × 1.8. At histological examination, the majority of the brain lesions (*n* = 36, 62.1%) were classified as WHO grade IV and 67.2% were IDH *wild-type*. At the visual examination, almost all patients (*n* = 54, 93.1%) showed increased tracer uptake, while four patients were negative and resulting in diffuse astrocytomas at neuropathology. Overall median TBR_max_ in the whole group was 4.51 and the median PET volume was 31.8 mL. Notably, WHO grade IV gliomas showed higher TBR_max_ and PET volumes as compared with WHO grade III and II lesions, as well as a trend towards a higher TBR_max_, although not at a statistically significant level, was observed in IDH *wild-type* gliomas with respect to IDH-mutant ones in the overall group. However, by stratifying patients according to each WHO grade, no significant differences in uptake (SUV, TBR) among *wild-type* and mutant gliomas were identified. In addition, no correlation was registered between [^18^F]-GE-180-related uptake parameters and other investigated molecular biomarkers (i.e., MGMT methylation status and TERT promoter mutation).

Quach and collaborators assessed the relationship between [^18^F]-GE-180 PET TSPO PET-derived parameters and survival in 88 patients affected by recurrent gliomas [28]. In particular, the authors assessed whether or not the intensity of the TSPO signal at recurrence was associated with post-recurrence survival (PRS) and time-to-treatment failure (TTF). The authors found a correlation between TSPO ligand uptake (SUVmax) at recurrence and tumor grade, with no significant difference between IDH *wild-type* and mutant lesions. Notably, in the sub-group of IDH-mutant gliomas, a lower SUVmax was associated with a more favorable outcome (i.e., longer PRS and TTP), thus suggesting a potential of the tracer to stratify patients with homogeneous molecular profiling. In the multivariate analysis, including imaging and clinical variables (e.g. WHO grade, IDH status, age, etc.), TSPO SUV_max_ remained an independent predictor of PRS. PET-derived volumes measured on [^18^F]-GE-180 images poorly correlated with volumes delineated on contrast-enhanced (ce)-MRI and, when available, on [^18^F]-FET PET.

### 3.3. Comparative Studies

Some studies compared the diagnostic performance of TSPO PET in gliomas with respect to that of other imaging modalities (MRI or amino-acid PET). Quantitative results of TSPO vs. amino-acid PET, when available, are reported in Table 2. In a first pilot study, Albert et al. compared the diagnostic accuracy of TSPO PET with [^18^F]-GE-180 with respect to that of MRI for the delineation of both newly diagnosed (n = 7) and post-therapy recurrent (n = 4) IDH *wild-type* GBM [22]. All the participants were first selected for TSPO-affinity status by DNA sequencing. In all cases, both MRI and PET/CT were carried out within a median time interval of 14 days. Several PET-derived parameters were calculated (SUV and TBR_max_), and tumor volumes of TSPO-positive lesions were measured by applying various SUV-based thresholds (SUV_BG_ × 1.6, SUV_BG_ × 1.8, SUV_BG_ × 2.0), thus generating corresponding volumes V_1.6_, V_1.8_, and V_2.0_. Afterward, MRI-based volumes were obtained using dedicated software (Oncentra MasterPlan^®^, Elekta Ltd., Crawley, UK) and compared with those obtained by using PET analysis. In all GBM patients, TSPO PET results were positive with high tumor-to-background contrast (median TBR_max_ = 6.61), while a very low signal was found in the normal brain. Although [^18^F]-GE-180 was found less sensitive to the genetic polymorphism with respect to the first- and second-generation TSPO ligands, the TBR_max_ value was greater in high-affinity and medium-affinity binders but not statistically significant. In PET-based volumes, regardless of the employed segmentation threshold, results were meaningfully greater than the corresponding MRI-based volumes. As a matter of fact, TSPO tracer uptake was also found in the GBM area with no CE on MRI.

Unterrainer and coworkers compared [^18^F]-GE-180 TSPO PET, [^18^F]-FET PET, and ce-MRI in 20 consecutively enrolled patients with newly diagnosed (n = 8) or recurrent (n = 12) HGGs and correlated imaging findings with both the histological tumor grade (according to the WHO 2016 classification) and molecular profiling [23]. After having tested all participants for genetic polymorphism, TSPO PET was carried out 60–80 min p.i. and the respective summed images were used for data analysis. In addition, PET/CT with [^18^F]-FET was obtained by a dynamic acquisition (0–40 min p.i.) and MRI was performed as a part of patients’ standard diagnostic work-up. On both TSPO and amino-acid PET images, several parameters were calculated: mean background activity (BG); TBR_max_; and biological tumor volume (BTV) applying a threshold of 1.8 x BG. In addition, minimal time-to-peak (TTPmin) was calculated on [^18^F]-FET PET/CT, and volume of contrast-enhancement (VOL_CE_) was measured on MRI. Histology was obtained in all newly diagnosed and in eight out of twelve HGGs, while in four cases with recurrent lesions, the standard of truth was represented by response evaluation according to Response Assessment in Neuro-Oncology (RANO) working group criteria [29]. Median TBR_max_ results were higher on [^18^F]-GE-180 PET than in [^18^F]-FET (i.e., 4.58 vs. 3.89), although not significantly different (*p* = 0.062). Notably, at subgroup analysis IDH *wild-type* HGGs showed meaningfully (*p* = 0.008) higher TBR_max_ on TSPO PET than on amino-acid PET, while no relevant difference in tracer uptake among the two PET-imaging modalities was found in the case of IDH-mutant HGGs. No correlation was registered among [^18^F]-GE-180 PET-derived parameters and TTPmin obtained on [^18^F]-FET PET. BTV delineated both on [^18^F]-GE-180 and [^18^F]-FET images was greater than the VOL_CE_ drawn on MRI.

The potential of TSPO PET for the in vivo imaging of glioma TME was investigated by Zinnhardt and colleagues [25]. In particular, the authors correlated the results of TSPO PET/MRI performed by employing the second-generation tracer [^18^F]-DPA-714 with in-depth immunophenotyping carried out in specimens obtained from glioma stereotactic biopsies. In addition, all the nine included patients (newly diagnosed glioma, n = 7; recurrence, n = 2) underwent, as a part of their diagnostic routine examination, both MRI and PET/MRI with [^18^F]-FET. All patients were screened for TSPO-associated gene polymorphism by DNA sequence and low-affinity binders were not included in the study. At histological examination, four cases were LGGs and five results were HGGs. PET was acquired through a hybrid PET/MRI tomograph by dynamic acquisition in 40 min for [^18^F]-FET and in 60 min for [^18^F]-DPA-714, respectively. All patients with newly onset HGGs were positive for both [^18^F]-FET and [^18^F]-DPA-714 uptake, both TSPO and amino-acid PET were negative in one case, and the [^18^F]-FET result was positive and [^18^F]-DPA-714 negative in two cases (i.e., one grade III and one grade II oligodendroglioma). Notably, the intensity of the TSPO signal was slightly lower than that obtained with amino-acid PET at quantitative analysis. Concerning the capability of TSPO-PET to image glioma TME, a significant and strong correlation was identified between [^18^F]-DPA-714 uptake and the number and activation level of glioma-associated myeloid cells (GAMs), particularly in regards to tumor-infiltrating immunosuppressive myeloid-derived cell populations. Notably, PET volumes calculated on [^18^F]-DPA-714 image exceeded the corresponding volumes obtained on [^18^F]-FET images, with differential spatial distribution.

Kaiser and colleagues combined dual-tracer PET with [^18^F]GE-180/[^18^F]-FET and ce-MRI to investigate their correlation in 34 patients with newly diagnosed GBM [19]. TSPO and amino-acid PET were acquired and analyzed as described in the previously cited paper [30]. Contrast-enhanced images were normalized voxel-by-voxel with the pre-contrast T1-weighted MRI to generate relative CE values (rCE), used as a surrogate biomarker for BBB leakage. Concerning tumor molecular profiling, the majority of glioma results were IDH1/2 *wild-type*. A voxel-by-voxel correlation was carried out in TSPO PET, amino-acid PET, and T1-weighted MRI to assess differences in PET-signal intensity (i.e., TBR) within the tumor and to establish whether or not [^18^F]GE-180 uptake was correlated with BBB leakage and therefore partially or completely attributable to non-specific tracer accumulation. In this regard, the authors found an absent to moderate correlation between TBR measured on PET images (both TSPO and amino-acid) with respect to MRI-based rCE value. In particular, some areas with no CE at the MRI image showed intensely increased TSPO tracer incorporation (hotspots), thus supporting the assumption that [^18^F]GE-180 is incorporated even in areas with intact BBB. Notably, in 53% of cases, no overlap among [^18^F]GE-180 hotspots and MRI hotspots was registered. On the contrary, the voxel-wise correlation between the two PET-imaging modalities was strong, with a moderate overlap of hotspots.

### 3.4. Kinetics, Methodological, or Feasibility Studies

Su et al. assessed the kinetics of [^11^C]-(R)PK11195 in gliomas and surrounding brain structures in 22 patients, in comparison with 10 age-matched healthy controls [20]. Glioma patients were “therapy-naïve”: a high-resolution research tomograph was used for image acquisition, employing a ^137^Cs point source for scatter and attenuation correction. After i.v. tracer injection, a dynamic scan was acquired over 60 min in list mode as 18 time-frames. Lesions were drawn both on PET and MRI images and then reciprocally compared, furthermore, tissue time–activity curves (TACs) were extracted from tumor regions as well as grey matter (GM) and white matter (WM) of the brains. Notably, BP_ND_ parametric maps were obtained through supervised cluster and cerebellar input function both in glioma patients and controls. Imaging results were correlated with glioma neuropathological assessment, encompassing the histochemical determination of TSPO expression on glial and tumor cells in specimens obtained after surgery or stereotactic biopsy. From the analysis of tracer kinetics within tumors, three distinct types were identified: (1) GM-like (lesion kinetics similar to that observed in cerebellar GM; (2) WM-like (lesion kinetics comparable to that of cerebral WM; (3) mixed kinetics. While low-grade astrocytomas mainly showed WM-like kinetics, oligodendrogliomas presented both GM-like and mixed kinetics; however, kinetics results were independent of tumor grade. BP_ND_ parametric maps generated with supervised cluster input results were smaller than those obtained with cerebellar input. Notably, TSPO expression in tumor cells positively correlated with tumor grade.

An interesting methodological study was performed by Vettermann and coworkers with the aim of establishing the impact of rs6971 polymorphism on the in vivo binding of [^18^F]-GE-180 with the aim of identifying the most adequate pseudo-reference tissue for quantification studies [26]. In a mixed population of patients affected by both neurodegenerative and neuro-oncological disease, the authors included 30 glioma patients, of which 11 were genotypically high-affinity binders (HAB, 36.6%), 14 were medium-affinity binders (MAB, 45.6%) and 5 were low-affinity binders (LAB, 16.6%). Regions of interest were manually delineated on the frontoparietal and cerebellar areas, subsequently, the respective SUVs were calculated. The authors found that LABs showed lower uptake values with respect to MAB and HAB but no significant differences were identified among MAB and HAB. In addition, the authors validated both frontoparietal and cerebellar hemispheres as reference regions for the quantification of [^18^F]-GE-180.

Radiomics is an interesting emerging discipline, based on artificial-intelligence-derived algorithms, aimed to extract from images potentially useful data, namely “feature” data, undetectable to the naked eye and employed to build predictive models [31]. In a recently published paper, Zounek and collaborators investigated the use of harmonization techniques aimed to integrate data extracted from [^18^F]-FET and [^18^F]-GE-180 images obtained from different centers (i.e., different tomographs, reconstruction methods, tumor segmentations, etc.), preserving clinical relevant information. For each acquisition, the authors emulated a multicentric data collection by employing 10 reconstruction settings and 9 segmentation methods. The authors calculated the statistical robustness of extracted features before and after applying the ComBat harmonization algorithm. It is worth mentioning that the majority of image-extracted data presented relevant differences when the various methods of reconstruction and segmentation were applied. Notably, [^18^F]-GE-180-extracted features were found more sensitive to the applied reconstruction methods with respect to [^18^F]-FET. The use of ComBat harmonization successfully aligned feature distribution in 87% of cases.

## 4. Discussion

**Figure 3:**
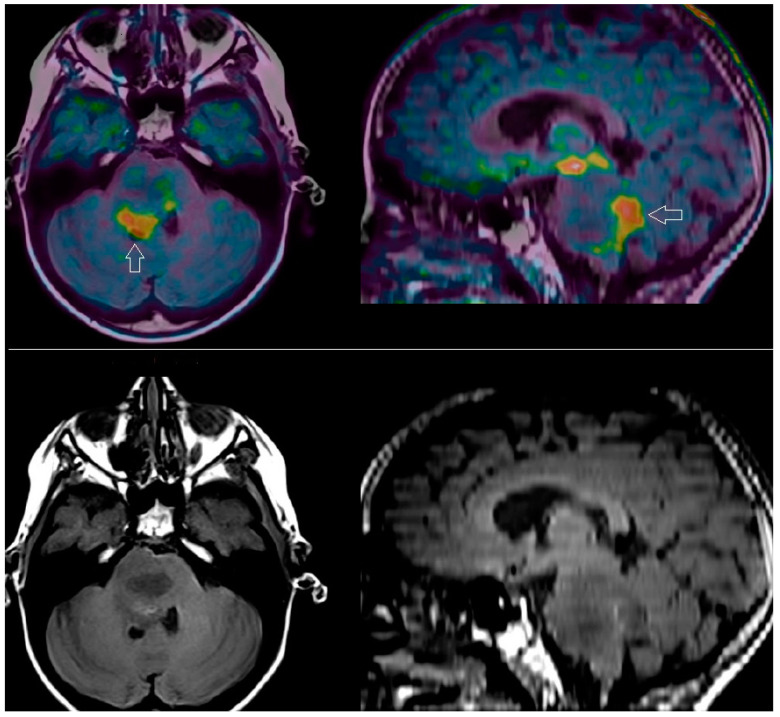
An 8-year-old female, diagnosed with diffuse intrinsic pontine glioma (DIPG), previously submitted to chemo-immunotherapy and radiotherapy. Amino-acid PET/MRI (post-processing co-registration) with [^18^F]-DOPA (upper row), carried out 8 months after RT demonstrated a rim of residual viable tumor tissue (arrow), evident on the axial (left) and sagittal (right) slices, within the posterior portion of the hypointense area revealed by MRI (lower row; axial on the left, sagittal on the right).

Tumors of the central nervous system represent a complex and heterogeneous category, encompassing numerous hystotypes graded and classified according to their biological and molecular characteristics [32]. Neuroimaging with MRI and amino-acid PET has a well-established role in clinical practice, as shown in Figure 3; nevertheless, it presents some limitations for the biological characterization of glioma and its TME.

TSPO has been found up-regulated in several cancer cells including glioblastoma but its role in promoting tumor growth and proliferation is still not clear. Pre-clinical studies have shown that increased TSPO levels are associated with enhanced tumorigenicity and cell proliferation rate [33]. Remarkably, emerging data are underlining the important role of mitochondria in cancer, as mutations in mitochondrial genes are frequently found in tumor cells, altering the mitochondrial bioenergetic and biosynthetic state [34]. As a matter of fact, in past years, ^99m^Tc-sestamibi has been widely used in clinical practice as a mitochondria-targeting imaging agent [35,36].

In this perspective, it might be plausible hypothesizing TSPO as a hallmark of glioma aggressiveness and propensity to evolve towards an anaplastic form. Although very preliminary, some of the cited studies [21,24] seem to point in this direction. In particular, a trend towards a higher grade of tracer uptake was found in gliomas characterized by the most aggressive molecular profiling (IDH *wild-type*). It has to be underlined, in this regard, that high-grade gliomas present a relevant recurrence rate after surgery and radio-chemotherapy. In such cases, as suggested by the report by Quach et al. [28], TSPO PET might be applied as a prognostic tool to identify patients with a less favorable prognosis, therefore requiring more careful monitoring or deserving further therapeutic options (i.e., second-line chemotherapy with regorafenib).

**Figure 4:**
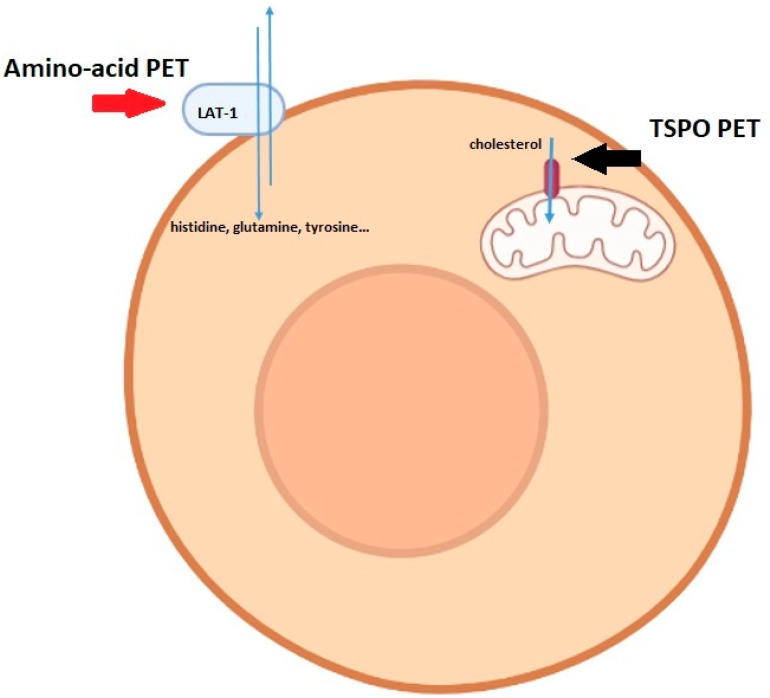
Schematic representation of the different locations in cells of the distinct targets for amino-acid and TSPO PET, respectively (figure created with BioRender.com).

Another relevant issue is represented by the role of TSPO PET with respect to amino-acid PET in the different clinical settings of glioma patients (diagnosis, recurrence, radiotherapy planning). First of all, it has to be highlighted that, although in the comparative study carried out by Zinnhardt’s group [25] the second-generation TSPO ligand [^18^F]-DPA-714 showed an inferior diagnostic performance in terms of signal-to-noise ratio with respect to [^18^F]-FET, the third-generation tracer [^18^F]-GE-180 presented excellent kinetics and image contrast, with superior TBR value in comparison to amino-acid PET [23,25]. Most interestingly, tumor volumes drawn on TSPO PET were greater, with only a moderate correlation of the “hot spots”, than those delineated on amino-acid PET. These last findings suggest a complementary role of the two PET imaging modalities (TSPO and amino-acid) for glioma characterization and are in agreement with previously published preclinical data. Cai and coworkers, in fact, found that the amino acid transporter light chain L system (LAT1) and TSPO are both overexpressed in glioblastoma preclinical models but the LAT1 expression was restricted to tumor cells, while TSPO was also expressed on microglia, tumor-associated macrophages, endothelial cells, and pericytes [37]. However, the clinical impact of volumes delineated on TSPO PET with respect to those drawn on amino-acid PET in radiotherapy planning and patients’ outcome is still unclear and will be a topic of future investigation. Figure 4 shows the different locations of biomarkers associated with amino-acid and TSPO PET.

TSPO PET provides the unique opportunity to explore glioma and its TME, thus paving the ground for patients’ selection for molecularly targeted therapies. Since an increased amount of tumor-infiltrating immunosuppressive cells (i.e., GAMs) were found to correlate with a more intense TSPO signal, this imaging modality might be applied to select patients suitable for GAMs-targeted immunotherapies, currently under investigation [38]. On the other hand, TSPO might be exploited for theranostic approaches, employing a couple of radiopharmaceuticals with similar or identical chemical properties and both directed towards a tumor-associated biomarker, the first labeled with radionuclide-emitting energy suitable for imaging and the other one conjugated with radioisotope-emitting particles to exert anti-tumor effects [39,40,41].

In spite of the aforementioned encouraging results, some issues concerning TSPO PET for imaging gliomas remain to be solved. First, the specificity of third-generation [^18^F]-GE-180 binding to glioma cells has been questioned, since it has been postulated that its incorporation might be mainly dependent on the grade of BBB disruption, more pronounced in HGGs [42]. However, the recently published paper by Kaiser et al. [19] seems to point in the opposite direction, indicating minimal or no correlation between MRI-based contrast enhancement and the areas of [^18^F]-GE-180 uptake in tumors, thus supporting a specific mechanism for the incorporation of the aforementioned tracer.

Second, although PET/MRI has been emerging as the most promising hybrid imaging modality in brain tumors [43], only one among the selected studies [25] employed a PET/MRI tomograph. Thus, the potential impact of PET/MRI with respect to PET/CT in this specific field is worthy of further study.

Finally, it has to be underlined that the majority of the selected studies have been carried out in Germany by a unique group of researchers and included cohorts with small sample sizes. From the perspective of widespread clinical use of TSPO PET, radiopharmaceutical availability, cost, and supplementation will play essential roles.

## 5. Conclusions

Initial clinical applications of TSPO PET in gliomas are interesting, particularly concerning the potential for its following applications: (1) in vivo assessment of glioma biological aggressiveness; (2) evaluation of glioma-associated TME, potentially amenable to molecularly targeted therapies; (3) prediction of survival after recurrence. Further studies, entailing multi-national cooperation and encompassing larger series, are needed to further investigate and support these encouraging preliminary findings.

## Figures and Tables

**Table 2:** Semiquantitative analysis in comparative studies between TSPO and amino-acid PET.

References	Type of Comparison	TSPO Ligand	TSPO PET Values (Median)	Amino-Acid PET Tracer	Amino-Acid PET Uptake Values (Median)	*p* Value *	Findings
Unterrainer et al. [23]	TSPO vs. amino-acid PET and MRI	[^18^F]-GE-180	TBR_max_ = 4.58 BTV = 31.1 mL	[^18^F]-FET	TBR_max_ = 3.89 BTV = 19.3 mL	0.062 0.062	TSPO and amino-acid PET provided different parameters in HGGs according to IDH mutational status, with partially overlapping volumes, greater than those obtained with MRI.
Zinnhardt et al. [25]	TSPO vs. amino-acid PET and MRI	[^18^F]-DPA-714	SUVmax/SUVmean = 2.5 SUVmean/SUVmean = 1.4	[^18^F]-FET	SUVmax/SUVmean = 2.8 SUVmean/SUVmean = 2.1	n.a.	TSPO and amino-acid PET provided complementary information in glioma. TSPO PET-derived parameters correlated with the amount of glioma-infiltrating immunosuppressive cells.

TBR: tumor-to-background ratio; SUV: standardized uptake value; IDH: isocitrate dehydrogenase; *: statistical significance was defined as two-tailed *p*-values < 0.05; n.a.: not available.

## Data Availability

Not applicable.
